# Léiomyosarcome vésical traité par cystectomie laparoscopique avec entérocystoplastie de remplacement: à propos d’un cas

**DOI:** 10.11604/pamj.2017.26.26.11189

**Published:** 2017-01-18

**Authors:** Dimitri Mbethe, Said Moudouni, Zacharia Dahami, Mohamed Amine Lakmichi, Ismael Sarf

**Affiliations:** 1 Service d’Urologie du Centre Hospitalo-Universitaire Mohammed VI de Marrakech, Maroc

**Keywords:** Léimyosarcome vesical, cystectomie, entérocystoplastie, laparoscopie, Leiomyosarcoma of the urinary bladder, cystectomy, enterocystoplasty, laparoscopy

## Abstract

Notre travail vise à rapporter un nouveau cas de léiomyosarcome vésical qui est une tumeur rare, et proposer une nouvelle approche thérapeutique compte tenu du caractère non consensuel de son traitement, cela à travers l'observation d'une patiente âgée de 31 ans, sans antécédent particulier, ayant consulté pour hématurie totale caillotante. Le bilan clinique et paraclinique, mettait en évidence une volumineuse masse tumorale solide occupant le dôme vésical et s'étendant à la paroi latérale droite, d'allure infiltrante, sans envahissement ganglionnaire ou organique local ou à distance. Notre prise en charge chirurgicale a consisté en une cystectomie totale par voie coelioscopique, avec entérocystoplastie de remplacement. Les suites opératoires ont été simples. Les contrôles radiologiques effectués à 3, 6, 12 et 24 mois ne montraient aucune récidive. Ainsi, une chirurgie adaptée peut être proposée selon les cas de figure, en vue d'améliorer la qualité de vie des patients présentant cette affection.

## Abstract (en)

Our study aims to report a new case of leiomyosarcoma of the urinary bladder, a rare tumor, and to propose a new therapeutic approach, given the non-consensual nature of its treatment. A 31-year old patient, with no particular previous history, presented with total haematuria with clot formation. The clinical and paraclinical assessment showed a voluminous solid tumor mass occupying the apex of urinary bladder and extending to the right lateral wall, with infiltrating appearance, without lymph node involvement or invasion of local or distal organs. Surgical management was based on total laparoscopic cystectomy and bladder replacement by enterocystoplasty. The postoperative course was uneventful. Radiological examinations at 3, 6, 12 and 24 months showed no recurrence. Thus, adaptive surgery should be proposed on a case-by-case basis, to improve the quality of life of patients suffering from this condition.

## Introduction

Le léiomyosarcome (LMS) vésical est une tumeur maligne rare. Son traitement reste encore non codifié. Nous présentons un cas survenu chez une jeune femme célibataire sans enfant.

## Patient et observation

**Figure 1:**
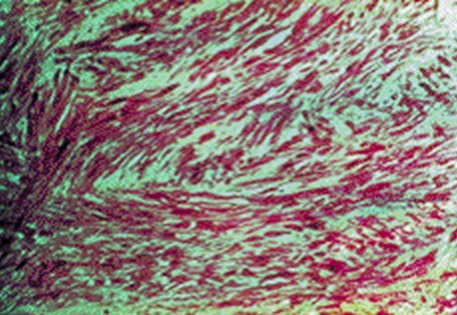
Léiomyosarcome myxoide grade II-III

**Figure 2:**
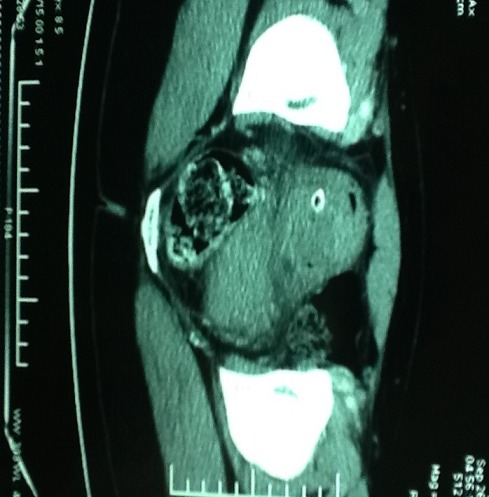
Uroscanner montrant la tumeur vésicale

**Figure 3:**
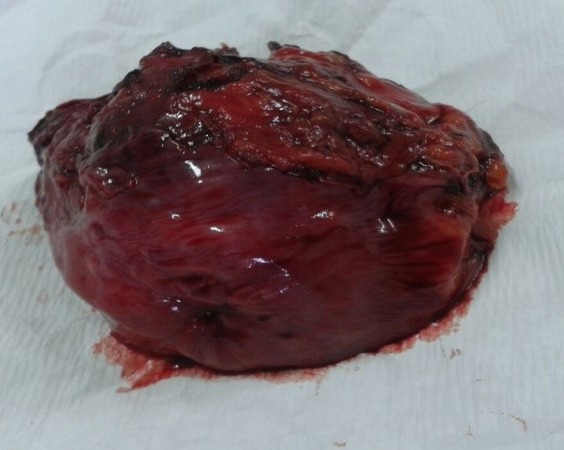
Pièce opératoire

**Figure 4:**
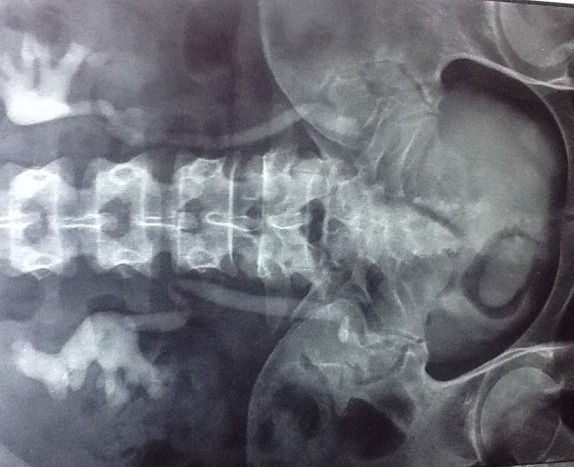
L’urographie intraveineuse à 3 mois post-opératoire

**Figure 5:**
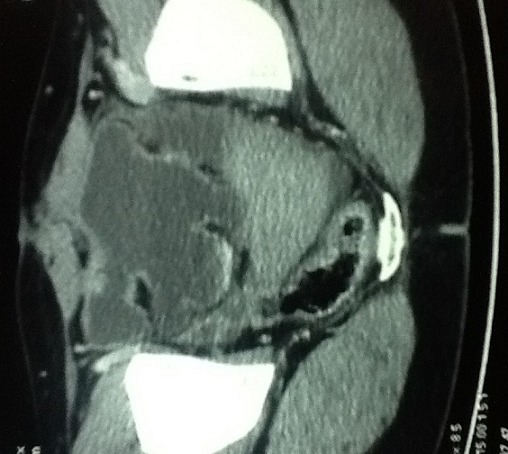
Scanner thoraco-abdomino-pelvien à 6 mois post-opératoire

Une patiente de 31 ans, sans antécédent, non connue tabagique consultait pour hématurie totale caillotante, évoluant depuis un mois dans un contexte d'altération de l'état général. A l'admission, on notait une pâleur conjonctivale, des parois vaginales souples sans masse perceptible au toucher pelvien. L'échographie abdomino-pelvienne montrait une masse vésicale irrégulière, hétérogène, de la paroi latérale droite mesurant 50 x 43 mm. La cystoscopie montrait une volumineuse masse tumorale solide, du dôme vésical s'étendant à la paroi latérale droite d'allure infiltrante. L'analyse histologique de la biopsie tumorale faite, montrait un enchevêtrement de faisceaux tourbillonnants et anastomotiques, d'orientation anarchique, à composante cellulaire superposée et de direction chaotique, avec des atypies fréquentes et des mitoses nombreuses, en faveur d'un processus sarcomateux fusiforme diversement myxoide et richement vascularisé: LMS myxoide grade II-III (Figure 1). Le scanner thoraco-abdomino-pelvien (Figure 2) ne montrait aucune métastase locale ou à distance. Le traitement consistait en une cystectomie totale coelioscopique avec entérocystoplastie de remplacement. L'analyse anatomopathologique de la pièce opératoire (Figure 3) confirmait le diagnostic avec des recoupes urétérales et urétrales saines. Les suites opératoires étaient simples. Aucune chimiothérapie adjuvante n'était préconisée, après avis des oncologues. L´urographie intra veineuse ou UIV (Figure 4) et la débimétrie réalisées au 3^ème^ mois montraient une bonne capacité néo-vésicale et un résidu post mictionnel peu significatif. Les scanners de control du 6^ème^ (Figure 5), 12^ème^ et 24^ème^ mois post-opératoires ne montraient aucune récidive tumorale.

## Discussion

Le léiomyosarcome de la vessie est une tumeur maligne qui se développe aux dépens du muscle lisse de la vessie. C'est une tumeur rare et elle représente 20% des tumeurs non épithéliales de la vessie. Elle survient aussi bien chez l'enfant que chez l'adulte, avec une incidence maximale après 60 ans. Depuis sa première description faite il y a un siècle par Gusshaver; environ une centaine de cas de LMS vésical ont été rapportés dans la littérature [1, 2]. L'étiologie de cette tumeur vésicale reste inconnue toutefois, pour certains auteurs des pathologies génétiques tel que la maladie de Von Recklinghausen, une radiothérapie au long cours sur la vessie, ou certaines chimiothérapies à base de cyclophosphamide pourraient favoriser sa survenue [3–5]. Le tableau clinique est souvent dominé par une hématurie souvent massive [1], ce fut le cas dans notre observation. Elle peut être associée à d'autres symptômes urinaires. A la cystoscopie, c'est une masse nodulaire lisse ou multilobée, aux limites nettes siégeant souvent au niveau du trigone expliquant le retentissement précoce sur le haut appareil [6]. Dans notre cas, la tumeur siégeait plutôt au niveau du dôme vésical, donc à distance des uretères, expliquant l'absence de retentissement sur le haut appareil urinaire. Le traitement du LMS vésical est essentiellement chirurgical, mais encore non codifié. Il repose sur une cystectomie partielle, pour les petites tumeurs superficielles [7] et une cystoprostatectomie radicale chez l'homme ou une pelvectomie antérieure chez la femme en cas de tumeurs infiltrantes. Certains auteurs préconisent une urètrectomie au cours du geste [8] et une chimiothérapie adjuvante à base de doxorubicine et du cis platine essentiellement dans les tumeurs infiltrantes et en cas d'envahissement ganglionnaire, d'autres la réservent pour les formes métastatiques [9, 10]. Chez notre patiente nous avons plutôt réalisé une cystectomie totale épargnant ainsi les organes génitaux internes, compte tenu du terrain (célibataire sans enfant) et vue son jeune âge (point de vue esthétique), la voie coelioscopique a été préférée, et une entérocystoplastie de remplacement a été réalisée. Notre patiente n'a pas bénéficié de chimiothérapie vue le bilan d'extension loco-régional négatif et après avis oncologique. L'évolution a été favorable avec un recul de 12 mois, sans récidive, sans métastase.

## Conclusion

En dépit du caractère hautement malin du LMS vésical une chirurgie adaptée sélective, peut néanmoins être proposée aux patients selon les cas de figure et permettre une qualité de vie satisfaisante après chirurgie.
